# Induction of Heme Oxygenase I (HMOX1) by HPP-4382: A Novel Modulator of Bach1 Activity

**DOI:** 10.1371/journal.pone.0101044

**Published:** 2014-07-14

**Authors:** Otis C. Attucks, Kimberly J. Jasmer, Mark Hannink, Jareer Kassis, Zhenping Zhong, Suparna Gupta, Sam F. Victory, Mustafa Guzel, Dharma Rao Polisetti, Robert Andrews, Adnan M. M. Mjalli, Matthew J. Kostura

**Affiliations:** 1 TransTech Pharma LLC, High Point, North Carolina, United States of America; 2 Biochemistry Department and Life Sciences Center, University of Missouri, Columbia, Missouri, United States of America; 3 Division of Biological Sciences, University of Missouri, Columbia, Missouri, United States of America; Chang Gung University, Taiwan

## Abstract

Oxidative stress is generated by reactive oxygen species (ROS) produced in response to metabolic activity and environmental factors. Increased oxidative stress is associated with the pathophysiology of a broad spectrum of inflammatory diseases. Cellular response to excess ROS involves the induction of antioxidant response element (ARE) genes under control of the transcriptional activator Nrf2 and the transcriptional repressor Bach1. The development of synthetic small molecules that activate the protective anti-oxidant response network is of major therapeutic interest. Traditional small molecules targeting ARE-regulated gene activation (e.g., bardoxolone, dimethyl fumarate) function by alkylating numerous proteins including Keap1, the controlling protein of Nrf2. An alternative is to target the repressor Bach1. Bach1 has an endogenous ligand, heme, that inhibits Bach1 binding to ARE, thus allowing Nrf2-mediated gene expression including that of heme-oxygenase-1 (HMOX1), a well described target of Bach1 repression. In this report, normal human lung fibroblasts were used to screen a collection of synthetic small molecules for their ability to induce HMOX1. A class of HMOX1-inducing compounds, represented by HPP-4382, was discovered. These compounds are not reactive electrophiles, are not suppressed by N-acetyl cysteine, and do not perturb either ROS or cellular glutathione. Using RNAi, we further demonstrate that HPP-4382 induces HMOX1 in an Nrf2-dependent manner. Chromatin immunoprecipitation verified that HPP-4382 treatment of NHLF cells reciprocally coordinated a decrease in binding of Bach1 and an increase of Nrf2 binding to the HMOX1 E2 enhancer. Finally we show that HPP-4382 can inhibit Bach1 activity in a reporter assay that measures transcription driven by the human HMOX1 E2 enhancer. Our results suggest that HPP-4382 is a novel activator of the antioxidant response through the modulation of Bach1 binding to the ARE binding site of target genes.

## Introduction

The basic metabolism of a cell generates reactive oxygen species (ROS) which oxidize cellular lipids, proteins, and DNA leading to production of reactive electrophiles which can lead to deleterious consequences if not eliminated [1]. The production of ROS and reactive electrophiles is counterbalanced by a conserved, well-defined set of cellular pathways leading to increased expression of oxidative stress-responsive proteins that degrade ROS, clear reactive electrophiles and increase cellular glutathione. This adaptive program is largely controlled by two proteins: Kelch like-ECH-associated protein 1 (Keap1) and the transcription factor NFEL2L2 (Nrf2). The Keap1-Nrf2 system has evolved to respond to intracellular oxidative stress; in particular the generation of reactive electrophiles produced from oxidation of endogenous cellular constituents as well as xenobiotics [2]–[4]. In the absence of cellular oxidative stress, Nrf2 levels in the cytoplasm are maintained at low basal levels by binding to Keap1 and Cullin 3, which leads to the degradation of Nrf2 by ubiquitination [2], [5]–[9]. During periods of oxidative stress, as levels of reactive electrophilic metabolites increase, the ability of Keap1 to target Nrf2 for ubiquitin-dependent degradation is disrupted, thereby increasing Nrf2 protein levels and its transport into the nucleus, resulting in transcription of antioxidant response genes [5], [6], [8], [10], [11]. Nrf2 binds to antioxidant response elements (AREs) found in the promoters of over 200 anti-oxidant and cytoprotective genes including NAD(P)H dehydrogenase, quinone 1 (NQO1), catalase (CAT), glutamate-cysteine ligase (GCLC), aldoketoreductase family members, thioredoxin reductase (TXNRD1), and heme oxygenase-1 (HMOX1) [12]. Activation of the anti-oxidant response via the Keap1-Nrf2 pathway is considered to be protective in nearly every organ system [4], [13]–[15].

There is, however, another mechanism by which ARE-regulated genes are controlled and that is through Bach1, a transcriptional repressor that binds to ARE promoter elements resulting in suppression of Nrf2 activity. Bach1 regulates ARE gene expression by binding to the small Maf proteins and ARE sequences that are also separately bound by Nrf2 [16]–[18]. Natively, Bach1 is bound by its ligand, heme, which causes it to be displaced from the ARE, exported from the nucleus and degraded [19]–[22]. Bach1 and its ligand coordinate the overall intracellular levels of heme and iron with anti-oxidant gene expression [23], [24]. Genetic evidence indicates that Bach1 deletion leads to a significant level of protection in a wide variety of murine disease models [25]–[32]. These observations suggest that ARE-regulated genes may be controlled by an intracellular ligand independent of ROS generation, electrophilic reactivity or elevation of Nrf2 levels in the cell. The potential; therefore, exists to discover novel, small molecules that target Bach1 and thereby elevate expression of ARE-regulated genes.

It has been previously demonstrated that Bach1 derepression is required prior to Nrf2-dependent HMOX1 gene expression [33]–[34]. Based on these observations, we report the development of a cell-based screening strategy to identify compounds that specifically modulate the expression of HMOX1 in normal human lung fibroblasts. The use of endogenous HMOX1 protein expression as a readout allowed the identification of compounds that specifically derepress Bach1 and induce transcription of an Nrf2-responsive gene. The identified compounds are not electrophiles, do not deplete cellular glutathione or otherwise incite a cellular stress response. We confirmed that these compounds modulate Bach1 directly using chromatin immunoprecipitation and reporter assays.

## Materials and Methods

### Cell culture

Normal human lung fibroblasts (NHLF) were purchased from Lonza and maintained in FBM medium supplemented with 2% FCS plus the supplied FGM-2 SingleQuot components (insulin, hFGF-B, and antibiotic/antifungal agents). Cells were carried for a maximum of four passages and grown in large T-175 flasks (CoStar). HepG2 hepatocellular carcinoma cells were purchased from ATCC and maintained in DMEM media containing 10% FCS and antibiotics. Compounds were kept in DMSO stock and diluted to a final concentration of 1% DMSO in complete medium for treatment.

### Immunofluorescence

NHLF cells were grown in either 96-well Optilux plates (Falcon; 4,000 cells per well) or 384-well Optilix plates (2,500 cells per well) and allowed to attach overnight in complete FBM medium. Cells were then treated with compound for a specified period of time depending on experiment. Following compound treatment, HMOX1 protein was detected using indirect immunofluorescence. Cells were washed in phosphate-buffered saline (PBS) containing calcium and magnesium, fixed in 4% paraformaldehyde in PBS for 10 minutes, washed twice with PBS, and then permeabilized with 0.2% Triton-X100 in PBS for 5 minutes. Afterwards, cells were blocked in a PBS solution containing 5% bovine serum albumin (BSA) and 0.05% Triton-X100. Cells were first probed with a primary mouse monoclonal antibody against human HMOX1 (Abcam) diluted in PBS containing 1% BSA, 0.01% Triton X-100 for 1 hour, washed twice, and then probed with a secondary goat anti-mouse Alexa 488 antibody (Invitrogen) for 1 hour. Hoescht stain (Invitrogen) was included to identify cell nuclei. Stained cells were washed in PBS, and HMOX-1 was visualized using the InCell 2000 instrument (General Electric).

### ROS and glutathione detection

HepG2 cells plated in 96-well Optilux plates were treated with compound for 1 hour after which 5 µM of the FITC-labeled ROS detection agent CellROX (Invitrogen) was added to the medium per manufacturer's instructions. After 15 minutes, cells were washed 3 times with PBS and then visualized live using a GE InCell 2000 imager. Glutathione was determined using the GSH/GSSG-Glo Assay (Promega). Briefly, cells grown in 96-well tissue culture plates were exposed to compound for 4 hours after which cells were lysed with the provided Total Glutathione Reagent and luminescence was determined using a SpectraMax 384 plate reader (Molecular Devices). Percent ROS or glutathione was calculated using fluorescence intensity; a 3-sigma increase in signal over control (solvent only) was deemed positive.

### Gene silencing

Silencing RNA for Nrf2 (SI03246614), Keap1 (SI03246439), and Bach1 (SI04364269) genes were purchased from Qiagen. NHLF cells were plated in complete medium at 4000 cells/well in 96-well culture plates (BD Falcon) one day prior to silencing. A 4X solution of siRNA (80 nM) and SiLentFect transfection lipid (6.75 µl/ml) (BioRad, cat# 170-3360) in serum-free FBM media was prepared and incubated at room temperature for 20 minutes. The siRNA solution was then diluted 1∶4 directly into NHLF cells plated in complete FBM. Cells were incubated for 48 hours prior to compound treatment. Sequences for siRNA were as follows: Nrf2: Sense 5′ GGAUUAUUAUGACUGUUAA 3′, antisense 5′ UUAACAGUCAUAAUAAUCC 3′; Keap1: sense 5′ AGGAUGCCUCAGUGUUAAA 3′, antisense 5′ UUUAACACUGAGGCAUCCU 3′; Bach1: sense 5′ GGAGUAGUGUGGAGCGAGATT 3′, antisense 5′ UCUCGCUCCACACUACUCCTA 3′.

### QuantiGene II mRNA detection

Gene expression was determined using the QuantiGene II system from Affymetrix following the manufacturer's protocol. Briefly, NHLF cells were grown in 96-well CoStar tissue culture plates (4,000 cells per well) and either subjected to siRNA gene silencing or directly treated with compounds for 51hours in 100 µL complete medium per well. Cells were then lysed by adding 50 µL Lysis Buffer (provided). Following the provided protocol, a portion of the RNA-containing lysate (5–10 µl) was hybridized at 54 degrees C overnight to RNA specific magnetic capture beads in the presence of blocking buffers, proteinase K and preordered mRNA probe sets specific for the genes of interest: HMOX1, Nrf2, Keap1, Bach1, and GAPDH. With the aid of a magnetic plate holder, capture beads containing the hybridized mRNA were washed and incubated with provided labeling probes. The amount and intensity of the labeled beads were determined using a Luminex xMAP cytometric scanner (BioRad). Results were tabulated and plotted using JMP software.

### Chromatin immunoprecipitation

NHLF cells were grown on 150 mm BD Falcon Integrid dishes. Cells were either treated with siRNA (see above) for 48 hours and/or treated with compound for six hours. Cells were cross-linked by adding formaldehyde to a final concentration of 1% and rocked for 10 minutes at room temperature. Cross-linking was stopped by adding glycine to a final concentration of 125 mM and rocked at room temperature for 5 minutes. Cells were washed three times with ice-cold 1X PBS, scraped into 1 ml of phosphate buffered saline (PBS) containing protease inhibitors (1x G-Biosciences Protease *Arrest*, 200 µM Na_3_VO_4_, and 1 mM PMSF) and collected by centrifugation (700xg for 4 min). Cell pellets were resuspended in 1 ml cell lysis buffer [5 mM Pipes pH 8.0, 85 mM KCl, 0.5% NP-40] containing protease inhibitors and incubated for 10 min on ice. Nuclei were pelleted by centrifugation (5000 rpm for 5 min) and resuspended in 350 µl nuclear lysis buffer [50 mM Tris pH 8.1, 10 mM EDTA, 1% SDS] containing protease inhibitors. After 10 minutes on ice, the samples were sonicated using the following protocol: 2×30 seconds at 30% power, 2×30 seconds at 35% power, 2×30 seconds at 40% power, 2×30 seconds at 45% power. Samples were centrifuged at maximum speed for 10 minutes at 4°C and the supernatants transferred to new tubes and diluted 5-fold in ChIP dilution buffer [0.01% SDS, 1.1% Triton X-100, 1.2 mM EDTA, 16.7 mM Tris pH 8.1, 167 mM NaCl] plus protease inhibitors. Samples were pre-cleared with protein A agarose slurry containing 10 mg/ml of E. Coli tRNA for 30 min at 4°C. For the total input control, 20% of the total supernatant was saved and frozen at −80°C. The remainder was equally divided among four tubes and incubated with rotation overnight at 4°C with: no antibody, 2 µg Nrf2 antibodies (H-300, Santa Cruz sc-13032), 4 µg Bach1 antibodies (2 µg of R&D Systems AF5776 and 2 µg of C-20, Santa Cruz sc-14700), or 2 µg Pol II antibodies (CTD4H8, Santa Cruz sc-47701). Immune complexes incubated with protein A agarose slurry containing tRNA for 1 hr at 4°C with rotation. Beads were collected by centrifugation at 4000 RPMs for 5 minutes. Beads were washed consecutively for 5 minutes on a rotating platform with 1 ml of each of the following solutions: low salt wash buffer [0.1% SDS, 1% Triton X-100, 2 mM EDTA, 20 mM Tris pH 8.1, 150 mM NaCl]; high salt wash buffer [0.1% SDS, 1% Triton X-100, 2 mM EDTA, 20 mM Tris pH 8.1, 500 mM NaCl]; LiCl wash buffer [0.25 M LiCl, 1% NP40, 1% deoxycholate, 1 mM EDTA, 10 mM Tris pH 8.0]; followed by a wash in TE Buffer. After each wash, beads were collected by centrifugation at 4000 RPMs for 5 minutes and supernatant was discarded. Complexes were eluted by adding 250 µl of elution buffer [1% SDS, 0.1 M NaHCO3] to pelleted beads and vortexed for 30 minutes. Samples were centrifuged at 14,000 rpm for 3 minutes and supernatant transferred to clean tubes. Elution was repeated and combined. Formaldehyde crosslinks were reversed by adding 1 µl of 10 mg/ml RNase and NaCl to a final concentration of 0.3 M and incubation at 65°C for 4–5 hours. To precipitate DNA, 2.5 volumes of 100% ethanol was added and the samples incubated overnight at −20°C. DNA was pelleted by centrifugation at max speed for 30 minutes at 4°C. The DNA was resuspended in 100 µl of water and 2 µl of 0.5 M EDTA, 4 µl 1 M Tris pH 6.5 and 1 µl of 20 mg/ml Proteinase K were added to each sample and incubated overnight at 45°C. DNA was purified using Thermo Scientific GeneJet PCR purification kit and eluted from the column in 50 µl of sterile dH_2_O. All chromatin immunoprecipitations were quantified using quantitative PCR.

### Quantitative PCR and data analysis

All quantitative PCR was carried out on an Applied Biosystems 7500 Real Time PCR System. Quantitative PCR was conducted in triplicate in an Applied Biosystems MicroAmp Optical 96-well Reaction Plate with a 25 µl reaction volume containing 12.5 µl of Thermo Scientific Maxima SYBR Green/ROX qPCR Master Mix, 2 µl of purified DNA and a final primer concentration of 0.15 µM for both forward and reverse primers. Primer were ordered from Sigma and sequences were as follows: HMOX1 EN2 ARE Sense: 5′-CACGGTCCCGAGGTCTATT-3′, REV: 5′-TAGACCGTGACTCAGCGAAA- 3′ and HMOX1 Promoter FOR: 5′-CAGAGCCTGCAGCTTCTCAGA-3′ REV 5′- GGAAACAAAGTCTGGCCATAGGAC-3′. Quantitative PCR was represented as % Input. The DNA used in each sample was representative of .8% of the total chromatin collected (20% total chromatin x 4% used for each qPCR replicate). This is a dilution factor of 125. For this reason, the Input was adjusted for dilution by subtracting Log_2_(125) from the raw Ct value. Percent Input was calculated for each sample by the following calculation: 125*2∧(Adjusted Input – Ct(IP sample)). Procedure for calculating %Input from raw Ct values was obtained from Invitrogen. Error was reported as the standard deviation of %Input value triplicates.

### Western blotting

NHLF cells transfected with siRNA molecules were lysed in High Salt ELB lysis buffer [1 M Tris pH 8.0, 1% NP-40, 250 mM NaCl, 5 mM EDTA] supplemented with protease and phosphatase inhibitors (1x G-Biosciences Protease Arrest, 200 µM Na_3_VO_4_, and 1 mM PMSF). One-half volume of 3x Sample buffer [6.7% SDS, 160 mM Tris-HCl pH 6.8, .005% Bromophenol Blue dye, 8.3% glycerol, 15% 2-BME) was added to the lysates. Lysates were sonicated using a Fisher Scientific Sonic Dismembrator (Model 500) at 35% power for 30 seconds on ice and then boiled for 10 minutes. Lysates were separated via SDS-PAGE on a 12.5% Bis-Tris polyacrylamide gel and transferred onto nitrocellulose membrane. After blocking overnight at 4° in 5% non-fat dry milk in PBS/0.1%Tween-20, blots were probed with the appropriate primary antibody for Keap1 (Cell Signaling), Nrf2 (Santa Cruz Biotechnology), or β-Tubulin (Sigma Aldrich). Blots were then probed with an appropriate horseradish peroxidase-conjugated secondary antibody (αMouse: Jackson-Immuno Research, αRabbit: Santa Cruz Biotechnology). Immunodetection was performed using Millipore Western HRP substrate and developed in a Fujifilm Intelligent Dark Box using LAS-3000 software.

### Bach1 luciferase assay

Single DNA strand bearing three copies of the human Maf-recognition element (MARE) core motifs, 5′-CTAGCTGCTGAGTCATGCTGAGTCATGCTGAGTCATC 3′, and its complementary strand, 5′-TCGAGATGACTCAGCATGACTCAGCATGACTCAGCAG 3′, were synthesized and annealed through standard procedures. The generated DNA fragment was then subjected to NheI and XhoI digestion and cloned into the pGL3-Luc basic vector that had also been digested with the same restriction enzymes. The clone, pGL-MARE-Luc, was confirmed via DNA sequencing before being used in the luciferase reporter assay. A FLAG tag was introduced to the N-terminus of the human Bach1 gene by cloning the gene into a pFLAG-CMV-6c vector (Sigma). Cysteine-to-Alanine substitutions (C435A, C461A, C492A and C646A) in the CP motifs were achieved through site-directed mutagenesis using the QuikChange II Site Directed Mutagenesis Kit from Agilent Technologies. HepG2 cells in 100 mm cell culture dishes were transfected with pGL-MARE-Luc plasmid DNA along with plasmid carrying the human Bach1 gene or the empty vector pFLAG-CMV-6c using Fugene6 (Promega). Transfected cells were trypsinized and re-plated into 96-well plates 20–24 hours after transfection. Compounds were added to cells 5–6 hours later, and then incubated overnight. The transfected and compound-treated cells were then gently washed with PBS followed by the addition of Luciferase substrate (Steadyliteplus, PerkinElmer). The cells were incubated for 15–30 minutes at room temperature to allow complete cell lysis before determining luminescent levels using in an Envision plate reader.

## Results

### Screening HMOX1 protein expression in NHLF cells

**Figure 1:**
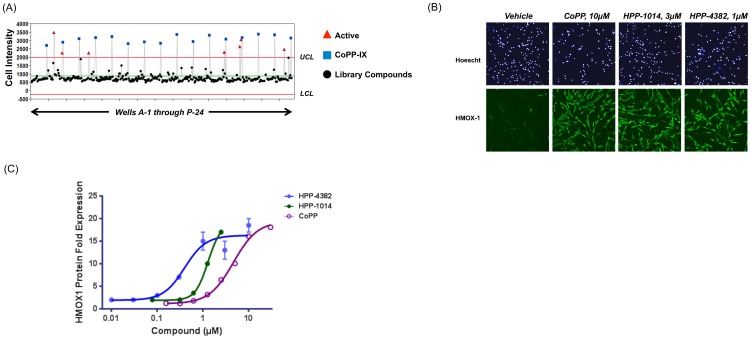
Identification of molecules that induce HMOX1 expression. (A) Human lung fibroblast cells were plated in 384-well Optilux plates and screened with compound libraries at 15 µM for 18 hours. Cells were then fixed, permeabilized, and probed with anti-HMOX1 antibody. Fluorescence intensity of HMOX1 staining was quantified with a GE InCell imager. Control charts were prepared using the statistical software JMP. HMOX1-staining intensities greater than the upper confidence limit were deemed hits. (B) Representative images of cells expressing HMOX1 following compound treatment. NHLF cells were cultured in 96-well Optilux plates as described in *Materials and Methods*. Cells were treated with indicated compound at selected concentrations for 18 hours after which HMOX1 expression was determined by immunofluoresence and quantified on a GE InCell imager. (C) Potency of CoPP, HPP-1014, and HPP-4382 were determined in NHLF cells. Cells were treated for 18 hours, after which they were fixed, permeabilized, and HMOX1 expression determined via immunofluoresence captured on a GE InCell imager.

Normal human lung fibroblasts (NHLF) cells grown in 384-well Optilux plates were treated with candidate compounds and incubated for 18 hours prior to fixation and staining with Hoescht dye and anti-HMOX1 antibody as described in *Materials and Methods*. Figure 1A provides representative data on performance of the assay; Cobalt Protoporphyrin IX (CoPP) was used as an internal positive control. The mean expression level and confidence limits of HMOX1 protein were estimated from the raw pixel intensities using JMP software (SAS Institute). Control charting was used to determine the relative ability of a compound to induce HMOX1. The global mean and variance of HMOX1 expression was estimated for all wells of the plate tested. From that, lower and upper confidence limits representing 3SD units above and below the mean are plotted. Values above the upper confidence limit indicate a well with a potentially active compound. As shown in Figure 1B, NHLF cells have a very low level of basal HMOX1 expression. Treatment with the positive control CoPP results in induction of HMOX1 protein as measured by specific immunofluorescence. Based on this method of compound activity classification, we identified a class of thiol-reactive (electrophilic) HMOX1 inducing compounds, exemplified by HPP-1014. In addition, a separate class of non-electrophilic yet potent HMOX inducing compounds, represented by HPP-4382, was discovered. The relative potency of the compounds was established using NHLF cells giving the rank order of potency as HPP-4382>HPP-1014>CoPP (Figures 1B, 1C). This rank order was maintained in HepG2 cells (Figure S1 in Data S1).

### HPP-4382 is not an electrophile, is not affected by N-acetylcysteine, and does not increase ROS

Chemical induction of Nrf2-dependent gene activation is often described as being driven by compounds with electrophilic groups. The chemical reactivity of these groups leads to alkylation of reactive thiols and generation of ROS. A key test of chemical reactivity is to incubate the compounds with a thiol-containing reductant. If the compound is reactive, a thiol-containing adduct will be formed that is detectable using mass spectrometry. Using this methodology, the chemical reactivity of HPP-4382 was compared to the electrophile bardoxolone-methyl (CDDO-Me) (Figure S2 in Data S2). Solutions of HPP-4382 and CDDO-Me were exposed to the thiol-containing reductants N-acetylcysteine (NAC), cysteine and dithiothreitol. CDDO-Me reacted with thiol groups as determined by detection of specific adducts by LC-MS (Tables S1 and S2 and Figure S3 in Data S2). Similar results are observed with HPP-1014 (data not shown). In contrast, no thiol-containing HPP-4382 adducts were detected, demonstrating that HPP-4382 is not thiol-reactive.

**Figure 2:**
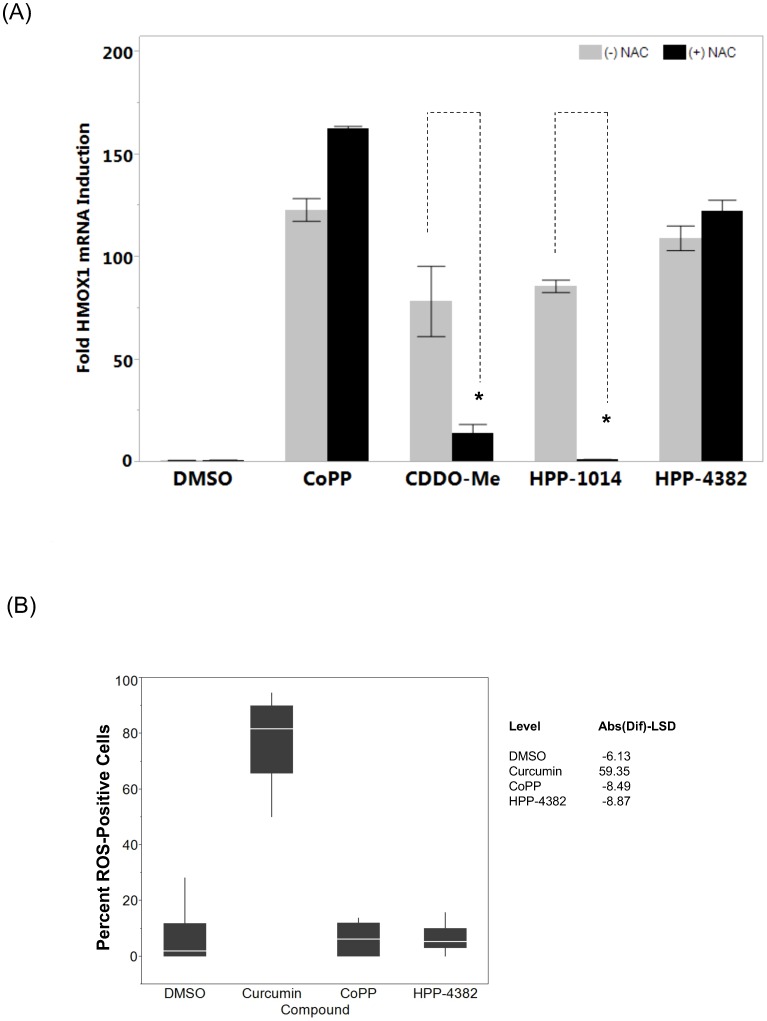
HPP-4382 is not a thiol-reactive electrophile. (A) Effect of N-acetylcysteine (NAC) on HMOX1 induction by HPP compounds. NHLF cells were pretreated with 5 mM NAC for one hour prior to treating with compounds for a further 5 hours (CoPP, 3 µM; CDDO-Me, 0.1 µM; HPP-1014, 3 µM; HPP-4382, 3 µM). Cells were then lysed and HMOX1 mRNA was detected using the Quantigene II method as described in *Materials and Methods*. *p<0.05, (B) Induction of reactive oxygen species (ROS) by HPP compounds in HepG2 cells. Cells attached to Optilux plates were treated with compound for 1 hour after which the FITC-labeled ROS-detecting agent CellROX was added for 15 minutes. The number and intensity of ROS-stained cells were captured with a GE InCell imager and the percentage of cells expressing ROS above a set threshold were determined; positive values show pairs of means that are significantly different. All samples in duplicate.

To assess thiol reactivity in cells, the ability of NAC to block HMOX1 induction was determined. NAC has been shown to suppress induction of Nrf2-dependent gene activation by electrophilic compounds, an attribute of both its chemical reactivity and its ability to maintain cellular glutathione levels. To test this premise, NHLF cells were treated with either CDDO-Me, the electrophilic compound HPP-1014, CoPP, or HPP-4382 in the presence or absence of 5 mM NAC. Both CoPP and HPP-4382 induced HMOX1 expression in the presence of NAC whereas induction of HMOX1 by both CDDO-Me and HPP-1014 was inhibited (Figure 2A).

Thiol-reactive electrophilic compounds often increase ROS levels in cells, as a consequence of their ability to deplete glutathione. Levels of ROS were measured in HepG2 cells following exposure to either HPP-4382 or curcumin, a highly reactive electrophilic compound. ROS levels, as measured by the proportion of cells that stained positive for CellROX, increased from an average of 8.1% in cultures treated with DMSO to 78% in cultures treated with curcumin. In contrast, at the highest tested dose of HPP-4382 (3 µM), ROS levels did not increase above background (6.4%; Figure 2B).

### HPP-4382 does not deplete cellular levels of glutathione

**Figure 3:**
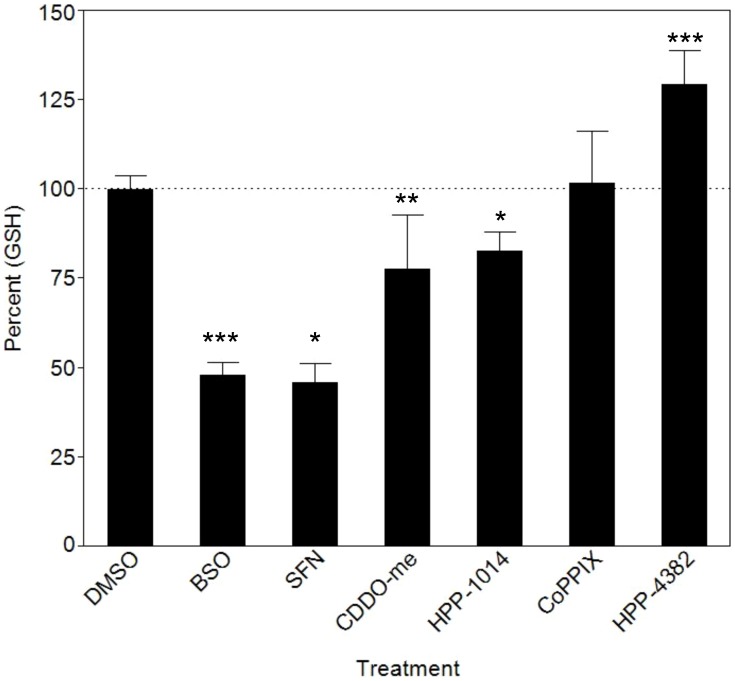
HPP-4382 increases cellular glutathione levels: NHLF cells grown in 96-well Optilux plates were treated with compounds for 4 hours (BSO, 200 µM; Sulphorafane, 10 µM; CDDO-Me, 0.1 µM; HPP-1014, 10 µM; CoPP, 10 µM; HPP-4382 3 µM) and glutathione levels were determined using the GSH/GSSG-Glo Assay Kit (Promega). Positive values show pairs of means that are significantly different. All samples in duplicate, error bars represent standard deviation compared to DMSO. *, *p*<0.05; **, *p*<0.01, ***; *p*<0.001.

Increased cellular ROS is often accompanied by a decrease in cellular glutathione levels. Glutathione was measured in NHLF cells following a 4-hour treatment with buthionine sulphoximine (BSO, an inhibitor of gamma-glutamylcysteine synthetase), electrophilic compounds including bardoxolone, sulforaphane and HPP-1014, and non-electrophilic compounds, including CoPP and HPP-4382. Glutathione levels were markedly reduced in cells treated with BSO (48%, p<.0001) or with the electrophilic compounds. However, neither CoPP nor HPP-4382 reduced cellular glutathione. In fact, cellular glutathione levels were significantly increased by HPP-4382 (129%, p = .0007) within four hours (Figure 3). Extended treatment of NHLF cells with all compounds revealed a recovery of cellular glutathione with all compounds except BSO (data not shown). The combination of a lack of ROS generation and increased levels of cellular glutathione suggest that HPP-4382 induces HMOX1 in a manner distinct from electrophilic activators of Nrf2.

### HPP-4382 induction of HMOX1 is Nrf-2 dependent

**Figure 4:**
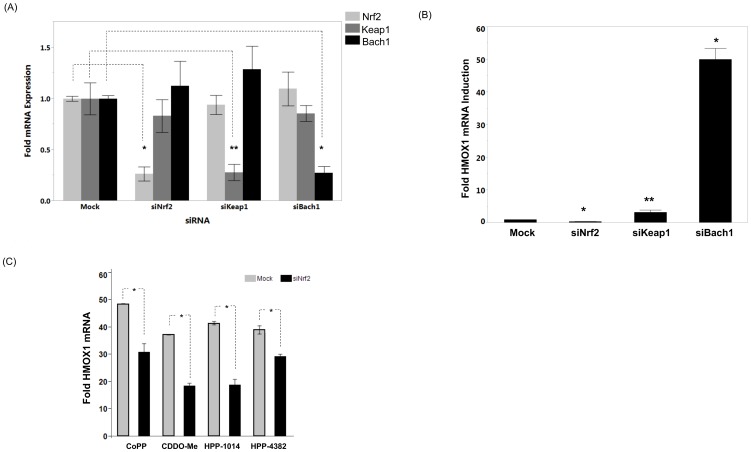
HMOX1 expression by HPP-4382 requires Nrf2. (A) NHLF cells were exposed to 20 nM per well each of Nrf2, Keap1 or Bach1 silencing RNA (or mock) for 48 hours as described in *Material and Methods*. Cells were then lysed and probed for transcription of Nrf2, Keap1, or Bach1 using the QuantiGene II RNA plex (Affymetrix). RNA expression was normalized to total GAPDH and expressed as fold induction over DMSO vehicle for the same gene. *, *p*<0.0001; **, *p*<0.05; all others not significant (*p*>0.1). (B) NHLF cells exposed to Nrf2, Keap1, or Bach1 siRNA or lipid-only vehicle for 48 hours were lysed and probed for transcription of HMOX1 using the QuantiGene II RNA plex. Detected HMOX1 RNA per well was normalized to total GAPDH in same well and shown as fold induction over DMSO;. *, *p*<0.0001; **, *p*<0.05. (C) HMOX1 gene expression in NHLF cells treated with Nrf2 siRNA. After silencing for 48 hours, cells were exposed to vehicle (DMSO) or compounds for 5 hours. HMOX1 and GAPDH RNA expression was determined using QuantiGene II mRNA quantitation. All samples were performed in duplicate, error bars represent standard deviation. *, *p*<0.05.

To determine if induction of HMOX protein expression by HPP-4382 remained dependent on Nrf2 despite being independent of ROS production, RNAi was used to reduce the expression of Nrf2, Keap1 and Bach1. NHLF cells were treated with siRNA to each gene, resulting in reduced mRNA levels for each gene by 73%, 72%, and 73%, respectively, as determined by the QuantiGene II mRNA plex (Figure 4A). Silencing of Nrf2 significantly decreased baseline levels of HMOX1, whereas silencing of Bach1 resulted in a 50-fold increase in expression of HMOX1 mRNA (Figure 4B). Keap1 silencing, which stabilizes Nrf2 protein levels (see below), only minimally elevated HMOX1 gene expression (approximately 3-fold). In NHLF cells transfected with siRNA against Nrf2 and subsequently treated with either the thiol-reactive compounds CDDO-Me or HPP-1014; or the non-reactive compounds CoPP or HPP-4382, there was a marked reduction in HMOX1 expression. (Figure 4C). Thus, maximal induction of HMOX1 by HPP-4382 is independent of ROS but is still dependent on the presence of Nrf2.

### HPP-4382 alters the balance of Nrf2 and Bach1 bound to the HMOX1 E2 ARE independent of Nrf2 and Keap1

**Figure 5:**
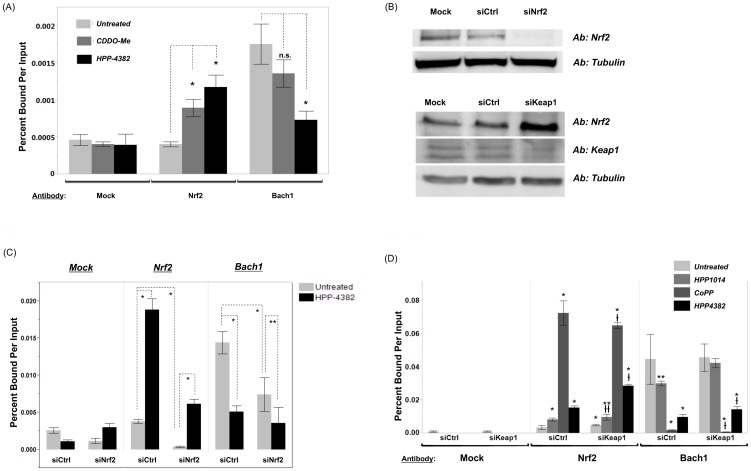
HPP-4382 alters occupancies of Nrf2 and Bach1 on the HMOX1 E2 promoter. (A) NHLF Cells were treated with 0.1 µM CDDO-Me or 1 µM HPP-4382 for 6 hours after which they were crosslinked with 1% formaldehyde in media, washed, and collected to be processed for chromatin immunoprecipitation as described in *Materials and Methods*. Precleared nuclear lysates were incubated with antibodies against Nrf2 or Bach1. Immune complexes were than isolated with E.coli tRNA/Protein A agarose beads, and the obtained purified DNA with subjected to qPCR using primers for HMOX1 E2 promoter. *, *p*<0.05 compared to the untreated sample of same antibody; n.s.  =  not significant. (B) NHLF cells were exposed to 20 nM Nrf2, Keap1, or control siRNA for 48 hours. Cells were lysed and separated via SDS-PAGE then Western blotted with antibodies against Nrf2, Keap1, or tubulin. (C) Cells transfected with either Nrf2 or control siRNA were subjected to chromatin immunoprecipitation after treatment with 1 µM HPP-4382 for 6 hours. Precleared nuclear lysates were probed with antibodies against Nrf2 or Bach1; a third set was not probed (mock). *, *p*<0.01; **, *p*<0.05. (D) Cells transfected with either Keap1 or control siRNA were subjected to chromatin immunoprecipitation after treatment with either 10 µM HPP-1014, 10 µM CoPP, or 1 µM HPP-4382 for 6 hours. Precleared nuclear lysates were probed with antibodies against Nrf2 or Bach1; a third set was not probed (mock). All samples were performed in triplicate, error bars represent standard deviation. *, *p*<0.01; **, *p*<0.05 compared to untreated siCtrl for same antibody probe. 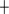, *p*<0.01; 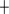 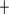, *p*<0.05 compared to untreated siKeap1 for same antibody probe.

Transcription of the HMOX1 gene is controlled, in part, through the binding of either Nrf2 or Bach1 to an ARE, termed HMOX1 E2, located approximately 9 kbp from the transcription start site. Chromatin immunoprecipitation was used to monitor Nrf2 and Bach1 occupancy at the HMOX1 E2 ARE. Under basal conditions, no significant differences in Nrf2 occupancy were observed at the HMOX1 E2 ARE. Following treatment of NHLF cells with either HPP-4382 or CDDO-Me, a 2- to 3-fold increase in Nrf2 occupancy was observed at the ARE. Under basal conditions, Bach1 occupancy was markedly higher than Nrf2 occupancy at the HMOX1 E2 ARE. HPP-4382, but not CDDO-Me, significantly reduced Bach1 occupancy at the HMOX1 E2 ARE (Figure 5A).

To provide insight into the mechanism whereby HPP-4382 is able to both increase occupancy of Nrf2 and decrease occupancy of Bach1, siRNA was used to reduce steady-state levels of either Nrf2 or Keap1 (Figure 5B). While siRNA knockdown of Nrf2 markedly decreased steady-state levels of Nrf2 protein, siRNA knockdown of Keap1 increased steady-state levels of Nrf2 protein as lack of Keap1-mediated degradation results in accumulation of Nrf2. Knockdown of Nrf2 decreased occupancy by Nrf2 at the HMOX1 E2 ARE (Figure 5C). HPP-4382 increased occupancy by Nrf2 in cells treated with control siRNA. In cells treated with both anti-Nrf2 siRNA and HPP-4382, HMOX1 E2 ARE occupancy by Nrf2 was also increased relative to the levels observed in cells treated with anti-Nrf2 siRNA only, but not to the level observed in cells treated with HPP-4382 without Nrf2 silencing (Figure 5C). Occupancy of the phosphorylated form of RNA polymerase II at the promoters of these genes paralleled Nrf2 occupancy at the corresponding ARE (data not shown).

Bach1 occupancy of the HMOX1 E2 ARE was reduced to approximately 50% of untreated control cells by the anti-Nrf2 siRNA. Bach1 occupancy of the HMOX1 E2 ARE was reduced to a greater extent, about 25% of untreated controls, in cells treated with both anti-Nrf2 siRNA and HPP-4382 (Figure 5C). Thus, reduction in Bach1 occupancy by HPP-4382 is not dependent on the presence of Nrf2. Instead, HPP-4382 reduces Bach1 occupancy of the ARE even when steady-state levels of Nrf2 are reduced by siRNA.

While anti-Nrf2 siRNA molecules decrease steady-state levels of Nrf2, anti-Keap1 siRNA molecules have the opposite effect of increasing steady-state levels of Nrf2 (Figure 5B). Thus the ability of siRNA-mediated knockdown of Keap1 to perturb occupancy by Nrf2 and Bach1 at the HMOX1 E2 ARE was determined. In general, Keap1 siRNA alone resulted in a modest increase in Nrf2 occupancy at the HMOX1 E2 ARE while Keap1 siRNA in combination with HPP-4382 resulted in a further increase of Nrf2 occupancy. Importantly, in the presence of anti-Keap1 siRNA, HPP-4382 was still able to decrease Bach1 occupancy to the same extent as treatment with HPP-4382 only (Figure 5D). Taken together, these results suggest that HPP-4382 induces changes in Bach1 occupancy regardless of steady-state levels of Nrf2.

The ability of HPP-4382 to alter occupancy of Nrf2 and Bach1 at the HMOX1 E2 ARE was compared to HPP-1014 and to CoPP (Figure 5D). HPP-1014 is expected to act through Keap1 to stabilize Nrf2, while CoPP is a mimetic of heme, a known ligand for Bach1 that reduces its steady-state levels [20]. Nrf2 occupancy at the HMOX1 E2 ARE was increased by HPP-1014 while Bach1 occupancy was only slightly reduced by HPP-1014 in the absence of anti-Keap1 siRNA. No reduction of Bach1 occupancy by HPP-1014 was observed in the presence of anti-Keap1 siRNA. In contrast, both HPP-4382 and CoPP markedly reduced Bach1 occupancy at the HMOX E2 ARE either in the absence or presence of anti-Keap1 siRNA. Thus, the pattern of altered Nrf2/Bach1 occupancy induced by HPP-4382 does not resemble the pattern induced by an electrophile but closely resembles the pattern induced by CoPP.

### Heme binding motifs are required for promoter activity by HPP-4382

**Figure 6:**
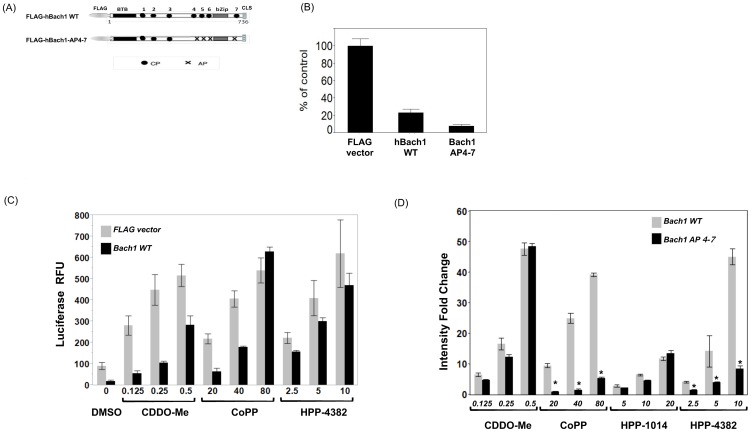
Heme binding motifs are required for activity of both CoPP and HPP-4382 on the HMOX E2 promoter. (A) Schematic representation of pFLAG-Bach1 (WT) and pFLAG-Bach1 (AP4-7) used in these experiments. (B) HepG2 cells were transfected with pGL-MARE-Luc plasmid DNA (containing the HMOX1 E2 promoter) plus a plasmid carrying either pFLAG-Bach1 (WT), pFLAG-Bach1 (AP4-7), or pFLAG-only for 24 hours. Cells were then transferred to 96-well plates and allowed to recover for 6 hours. After washing, Luciferase substrate was added for 30 minutes and fluorescence was measured on an Envision reader. (C, D) HepG2 cells were transfected and replated in 96-well plates as described in B, but treated with compounds at indicated concentrations (µM) overnight prior to determining luciferase activity. In (D), data is reported as fluorescence intensity fold over DMSO-treated cells in each set of transfection *, p<0.0001 compared to Bach1-WT expressing cells at same compound doses. Each sample was performed in quadruplicate, error bars represent standard deviation.

The ChIP experiments demonstrated the ability of HPP-4382 to elicit the removal of Bach1 from the HMOX1 promoter independent of Nrf2 steady-state levels. This suggests that HPP-4382 acts directly to modulate binding of Bach1 to AREs. To more fully explore the effects of compound on Bach1 activity, a luciferase reporter assay using the HMOX1 E2 ARE as a target was developed. Expression of HMOX1 E2-dependent luciferase expression was determined in HepG2 cells co-transfected with an HMOX1 E2-dependent reporter plasmid and a plasmid expressing FLAG-tagged wildtype Bach1 (Figure 6A). Luciferase expression was markedly lower in cells expressing Bach1, indicating effective repression of HMOX1 E2-dependent transcription by Bach1 (Figure 6B). To determine the ability of test compounds to activate HMOX E2-dependent expression in the presence of Bach1, cells were treated with CoPP, CDDO-Me, or HPP-4382. All three compounds were able to induce luciferase expression, demonstrating their ability to overcome Bach1 repression of HMOX E2-dependent transcription (Figure 6C).

The ability of hemin and CoPP to derepress Bach1 has been related to the presence of 4 CP motifs spanning the bZIP domain of the protein [21]. Mutation of these CP motifs markedly reduced heme binding to Bach1 and abrogated the ability of hemin to derepress Bach1. To probe the role of these CP motifs in modulation of Bach1 by HPP-4382, a mutant Bach1 protein containing Cysteine to Alanine substitutions at CP motifs 4 through 7 was constructed (Figure 6A). FLAG-hBach1-AP4-7 was more effective at repression of HMOX1 E2 ARE-dependent transcription than wild-type Bach1 (Figure 6B). Nonetheless, CDDO-Me and HPP-1014 still were able to activate HMOX1 E2-dependent luciferase expression in the presence of mutant Bach1 proteins, indicating that the CP motifs in Bach1 are not critical for efficient derepression of Bach1 by an electrophile (Figure 6D). In contrast, the ability of both CoPP and HPP-4382 to induce HMOX1 E2 ARE-dependent luciferase expression in the presence of the mutant Bach1 protein was sharply inhibited. The failure of CoPP to derepress FLAG-hBach1-AP4-7 is in line with observations demonstrating that these CP motifs are required for derepression of Bach1 by heme, and are essential components of a metalloporphyrin binding site in Bach1. That these CP motifs are also required for the ability of HPP-4382 to derepress Bach1 indicates a requirement for this metalloporphyrin binding site in Bach1 for induction of HMOX gene expression by HPP-4382.

## Discussion

In light of the widespread role of oxidative stress in the pathology of diverse human diseases and the ability of the Nrf2-dependent antioxidant response gene network to protect against oxidative stress, considerable effort has been directed towards discovering compounds that can increase the activity of Nrf2. Currently, all described small molecule inducers of Nrf2 activity are reactive electrophiles [13], [35], [36]. Typically, such compounds are not considered pharmaceutically acceptable as they can present safety and toxicity liabilities. Two such molecules, bardoxolone (CDDO) and dimethyl fumarate (DMF), have recently completed clinical trials. Both compounds are chemically reactive alkylating electrophiles. The intrinsic chemical promiscuity of Bardoxolone results in alkylation of a large number of proteins [37]. As a consequence, bardoxolone has a complicated pharmacological and toxicological profile with significant clinical safety problems. Similarly, dimethyl fumarate (DMF) is an electrophile that rapidly reacts with glutathione [38]–[40]. DMF, however, has not shown the same toxicities in humans as seen with bardoxolone. Given the rather divergent toxicology and adverse event profiles seen with bardoxolone and DMF, we conclude that induction of Nrf2 can be advantageous, but that the electrophilic character of the molecule is crucial and thus sets significant limitations on the safety and efficacy of such compounds.

An alternative approach to regulating Nrf2-dependent gene expression is through targeting the transcriptional repressor Bach1. Bach1 is a member of the BTB and CNC transcriptional regulator family that, like Nrf2, binds to ARE sequences as heterodimeric complexes with small Maf proteins [18] A major physiological role for Bach1 is in iron homeostasis through regulation of the expression of heme oxygenase-1 (HMOX1), ferroportin (FPN1) and Ferritin (FTH) genes [23], [24], [33], [41]. Elevation of intracellular hemin leads to induction of HMOX1 enzyme activity. Consequently, hemin is converted to carbon monoxide, bilirubin and free iron. As hemin levels are reduced, Bach1 is resynthesized and repression of HMOX1 and other genes is restored. Thus Bach1 coordinates the overall intracellular levels of hemin and iron metabolizing genes with anti-oxidant gene expression [19], [21], [22], [24].

The pharmacology of Bach1 modulation by heme and its metalloporphyrin mimetics has been examined in a variety of settings. Cobalt Protoporphyrin (CoPP) has been shown to have considerable pharmacological benefit in models of diabetes-linked vascular and renal damage [42]–[45], Ang II mediated hypertension [46], [47], renovascular hypertension [48], arterial thrombosis [49] and other oxidative stress-mediated pathologies. Inhibition of Bach1 itself has been suggested to be of benefit in diseases such as non-alcoholic steatohepatitis [50] and insulin resistance [51]. However, CoPP and most metalloporphyrins have limited bioavailability and therefore are unsuitable in most clinical settings. Thus, identifying molecules that can mimic the ability of metalloporphyrins to modulate Bach1 activity directly may have a high degree of therapeutic utility in a number of clinical settings without the potential liabilities of an electrophilic molecule.

Herein, we report the characterization of a novel molecule, HPP-4382, which induces HMOX1 in a manner distinct from other Nrf2 activators. We also demonstrated the ability of HPP-4382 to induce other Phase 2 genes, including NQO1 and TXNRD1 (Table S3 in Data S3). HPP-4382 does not have electrophilic properties, as determined by its structure and lack of chemical reactivity with common thiol-containing compounds such as N-acetylcysteine. To further characterize HPP-4382, we screened a selection of alternative genes for expression: two markers of endoplasmic- or general cellular stress, HSPA6 and GADD45A, and ICAM1, a target of NF-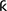
B. Using these orthogonal measures of cellular pathway analysis, we confirmed that HPP-4382, in contrast to bardoxolone, did not induce significant cellular stress at high doses as measured by HSPA6 expression and that the mechanism of HPP-4382 activity does not appear dependent on NF-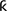
B as ICAM1 expression is not induced by HPP-4382 (data not shown).

It has been demonstrated that CoPP and hemin induce HMOX1 in an Nrf2-dependent manner through inhibition of Bach1 binding to HMOX1 promoter elements [21], [22], [33], [34]. Our data suggest that HPP-4382 functions to induce HMOX1 in a similar manner. We confirmed the role of Nrf2 in the regulation of HMOX1 gene expression by HPP-4382 using genetic silencing of Nrf2. Knockdown of Nrf2 expression resulted in reduced induction of HMOX1 by HPP-4382, CoPP and bardoxolone, consistent with the well-characterized role of Nrf2 as a critical activating transcription factor for HMOX1.

Pharmacological elevation of Nrf2 protein levels without concomitant derepression of Bach1 fails to induce HMOX1 [33]. Similarly, genetic silencing of Keap1 is insufficient to maximally activate HMOX1 gene expression in Keap1 null mice [52]. These data indicates the clear need for Bach1 derepression for HMOX1 gene expression. We probed this hypothesis in NHLF cells by silencing the three key components of the regulatory pathway. First, Bach1 silencing is sufficient to maximally induce HMOX1 mRNA expression, consistent with published results. On the other hand, Keap1 silencing resulted in significantly less HMOX1 induction in the absence of compound. Our results are consistent with the suggestion that Bach1 represents a dominant layer of control on HMOX1 expression in NHLF cells.

We further probed the ability of HPP-4382 to modulate transcription factor binding to the HMOX1 promoter via chromatin immunoprecipitation. In these experiments, HPP-4382 was compared to the electrophile CDDO-Me (Bardoxolone). Both compounds increased binding of Nrf2 at the HMOX E2 enhancer and binding of RNA polymerase II to the HMOX promoter, consistent with the ability of these compounds to activate HMOX1 transcription in an Nrf2-dependent manner. However, only HPP-4382, but not CDDO-Me, resulted in robust decreases in binding of Bach1 to the HMOX1 E2 enhancer element, suggesting that HPP-4382 has a mode of action distinct from that of CDDO-Me. To test this idea further, we altered steady-state levels of Nrf2 by gene silencing and measured occupancy of Bach1 at the HMOX1 E2 enhancer. In the presence of anti-Nrf2 siRNA, which significantly reduced steady state levels of Nrf2, Bach1 occupancy of the HMOX1 E2 enhancer was decreased by HPP-4382. In the converse experiment, when steady-state levels of Nrf2 were increased by gene silencing of Keap1, HPP-4382 was also able to decrease occupancy of Bach1 at the HMOX1 E2 enhancer. Thus, the ability of HPP-4382 to decrease binding of Bach1 to the HMOX1 E2 enhancer is independent of steady-state levels of Nrf2.

To further examine the mechanism by which HPP-4382 modulates Bach1, we created reporter assays controlled by the ARE element found in HMOX1-E2 and which is known to be regulated by Bach1. In addition, we created a modified Bach1 that is unable to respond to hemin and hemin mimetics, including CoPP. In these assays, both wild-type Bach1 and FLAG-hBach1-AP4-7 efficiently repressed basal levels of luciferase expression. CDDO-Me was able to derepress both the mutant and wild-type Bach1 proteins, resulting in increased levels of ARE-dependent gene expression. However, while CoPP efficiently derepressed the wild-type Bach1 protein, CoPP did not affect the repressive action of the mutant Bach1 protein. Similarly, HPP-4382 was able to overcome repression of ARE-dependent gene expression by wild-type Bach1 protein but not mutant Bach1 protein. Taken together, the results from the ChIP and derepression assays provide supporting evidence that HPP-4382 interferes with the ability of Bach1 to bind DNA. However, while heme has been reported to induce nuclear export and subsequent cytoplasmic degradation of Bach1, HPP-4382 does not appear to alter the steady-state levels or nuclear-cytoplasmic distribution of Bach1 (data not shown), suggesting that HPP-4382 may not fully mimic the action of heme as a ligand of Bach1. Nevertheless, the non-electrophilic character of HPP-4382 and the fact that an intact heme binding site in Bach1 is required for modulation of Bach1 activity indicates that HPP-4382 represents a first-in-class compound that is able to activate the anti-oxidant response gene network by specific modulation of Bach1 activity. We believe that this type of compound will provide therapeutic benefit in a variety of disease settings without the toxicities associated with electrophilic inducers of Nrf2 activity.

## Supporting Information

Data S1 **HMOX1 activation in HepG2 cells.**
(DOCX)

Data S2 **Comparison of electrophilic reactivity towards reduced glutathione.**
(DOCX)

Data S3 **Expression of Phase II genes in NHLF cells.**
(DOCX)
