# Supplementary material for: Induction of Heme Oxygenase I (HMOX1) by HPP-4382: A Novel Modulator of Bach1 Activity
Source: PLoS One. 2014 Jul 14;9(7):e101044. doi: 10.1371/journal.pone.0101044 (PMC4096395; doi:10.1371/journal.pone.0101044)
Supplement: Data S1 — HMOX1 activation in HepG2 cells. (DOCX) [file pone.0101044.s001.docx]

Data S1: HMOX1 activation in HepG2 cells

HepG2 cells were grown in 96-well Optilux™ plates (Falcon; 5,000 cells per well) and allowed to attach overnight in complete DMEM medium. Cells were then treated with compound for 18 hours. Following treatment, cells were washed in phosphate-buffered saline (PBS) containing calcium and magnesium, then fixed in 4% paraformaldehyde in PBS for 10 minutes, washed twice with PBS, and then permeabilized with 0.2% Triton-X100 in PBS for 5 minutes. Afterwards, cells were blocked in a PBS solution containing 5% bovine serum albumin (BSA) and 0.05% Triton-X100. Cells were probed with a primary mouse monoclonal antibody against human HMOX1 (Abcam) in PBS containing 1% BSA, 0.01% Triton X-100 for 1 hour, washed twice, and then probed with a secondary goat anti-mouse Alexa™ 488 antibody (Invitrogen) plus the nuclear stain Hoescht (Invitrogen) for 1 hour. Stained cells we washed in PBS and HMOX-1 visualized using the InCell© 2000 instrument (General Electric).

**Figure S1:** HMOX1 induction by CoPP, HPP-1014, and HPP-4382 in HepG2 cells. Cells were treated for 18 hours, after which they were fixed, permeabilized, and HMOX1 expression determined via immunofluoresence captured on a GE InCell imager.
