# Supplementary material for: Induction of Heme Oxygenase I (HMOX1) by HPP-4382: A Novel Modulator of Bach1 Activity
Source: PLoS One. 2014 Jul 14;9(7):e101044. doi: 10.1371/journal.pone.0101044 (PMC4096395; doi:10.1371/journal.pone.0101044)
Supplement: Data S2 — Comparison of electrophilic reactivity towards reduced glutathione. (DOCX) [file pone.0101044.s002.docx]

Data S2: Comparison of electrophilic reactivity towards reduced glutathione

The adduct formation through conjugate addition of a nucleophile (GSH) on CDDO-Me and any potential electrophilic reaction of HPP-4382 was followed at 26°C and at 40°C through HPLC/MS analysis (Tables S1, S2). A detailed evaluation of the reaction illustrates CDDO-Me reacts reversibly with GSH to form a more polar adduct both at 26°C and at 40°C (Figures S2, S3). No such adduct formation was observed with HPP-4382 under these reaction conditions.

Protocol for reaction between compounds (CDDO-Me and HPP-4382) and GSH

The compositions of stock solutions are as follows: Solution A: 20 mM CDDO-Me in PEG400; Solution B: 20 mM HPP-4382 in PEG400; Solution C: 75 mM GSH in water; Solution D: Phosphate buffer (100 mM, pH = 8.0):Acetonitrile (70:30, v/v)

Stock solutions A & B were diluted to 660 µM final concentration in stock solution D. GSH (10 eq.) was added and the reaction vials were stirred at 26°C and at 40°C. All reactions were analyzed using HPLC/MS at three time points (t = 0, 10 min, 0.5, 24 h). HPLC analysis was done using a Waters 1525 binary HPLC pump with UV detection at 254 nm on a Waters MUX-UV 2488 UV-VIS detector. Typically, a 3 min gradient was run from 25% B (97.5%acetonitrile, 2.5% water, 0.05% TFA) and 75% A (97.5% water, 2.5% acetonitrile, 0.05% TFA) to 100% B on Sepax GP-C18 (4.6 x 50 mm) column at 50°C. All mass spectra (MS) were obtained using electrospray ionization (ESI) on a Waters zqMUX with MassLynx 4.0 software.

CDDO-Me and HPP-4382 adduct formation with GSH

As demonstrated by HPLC analysis, CDDO-Me forms an adduct with GSH at 26°C and at 40°C within 10 min of reaction time (Table S1, Figure S2). This adduct clearly elutes as a separate peak at an earlier retention time (RT=1.24 min) compared to CDDO-Me (RT = 1.74 min). Further monitoring of the reaction reveals that the reaction does not go to completion suggesting the possibility of a reversible reaction. After 24 h CDDO-Me, adduct can still be detected using HPLC/MS at 26°C reaction; however, both CDDO-Me and CDDO-Me adduct decompose in reaction at 40°C^1^. HPP-4382 under these conditions does not show any adduct formation and the HPLC analysis shows a single peak at retention time (RT=1.30 min).

CDDO-Me adduct formation is further characterized through MS (ESI) analysis (Table S2, Figure S2). The calculated molecular weight for CDDO-Me and CDDO-Me adduct is 505.70 and 813.0 respectively. MS analysis demonstrates the observed mass (m/z) of (M^+^) 813.0 and 505.9 respectively for the peaks eluting at retention time (RT=1.24 and 1.74 min). Furthermore the calculated molecular weight for HPP-4382 is 456.96, and the observed mass in MS analysis (m/z) is (M^+^) 457.0 for the peak eluting at retention time (RT=1.25 min). Therefore the MS analysis reaffirms that CDDO-Me forms a polar adduct with GSH (Figure S3) whereas HPP-4382 does not form any adduct with GSH under these reaction conditions.

**Table S1: HPLC analysis of reaction between CDDO-Me and HPP-4382 with GSH**

| **HPP-ID** | **T = 0 min Observed Retention Time (min)** | | **T = 10 min Observed Retention Time (min)** | | **T = 0.5 h Observed Retention Time (min)** | | **T = 24h Observed Retention Time (min)** | |
| --- | --- | --- | --- | --- | --- | --- | --- | --- |
| **Temperature** | **26°C** | **40°C** | **26°C** | **40°C** | **26°C** | **40°C** | **26°C** | **40°C** |
| **HPP-4382**  **HPP-4382 Adduct** | **1.25**  **n.d.^a^** | **1.25**  **n.d.^a^** | **1.24**  **n.d.^a^** | **1.24**  **n.d.^a^** | **1.25**  **n.d.^a^** | **1.25**  **n.d.^a^** | **1.25**  **n.d.^a^** | **1.25**  **n.d.^a^** |
| **CDDO-Me**  **CDDO-Me Adduct** | **1.72**  **n.d.^a^** | **1.72**  **n.d.^a^** | **1.74**  **1.24** | **1.74**  **1.24** | **1.74**  **1.24** | **1.74**  **1.24** | **1.72**  **1.24** | **n.d.^a^**  **n.d.^a^** |

^a^ Not detected

**Table S2: MS analysis of reaction between CDDO-Me and HPP-4382 with GSH**

| **HPP-ID** | **Calculated Mol Wt.** | **T = 0 min Observed Mol Wt.** (M^+^) | | **T = 10 min Observed Mol Wt.** (M^+^) | | **T = 0.5 h Observed Mol Wt.** (M^+^) | | **T = 24h Observed Mol Wt (M^+^)** | |
| --- | --- | --- | --- | --- | --- | --- | --- | --- | --- |
| **Temperature** |  | **26°C** | **40°C** | **26°C** | **40°C** | **26°C** | **40°C** | **26°C** | **40°C** |
| **HPP-4382**  **HPP-4382 adduct** | **456.96** | **457**  **n.d.^a^** | **457**  **n.d.^a^** | **457**  **n.d.^a^** | **457**  **n.d.^a^** | **457**  **n.d.^a^** | **457**  **n.d.^a^** | **457**  **n.d.^a^** | **457**  **n.d.^a^** |
| **CDDO-Me**  **CDDO-Me Adduct** | **505.70 813** | **506**  **n.d.^a^** | **--- -**  **--** | **506**  **813** | **506**  **813** | **506**  **813** | **506**  **813** | **506**  **813** | **n.d.^a^**  **n.d.^a^** |

^a^ Not detected

**Figure S2: HPLC/MS analysis.** A) CDDO-Me at T = 0 min, 26°C B) HPP-4382 at T = 0 min, 26°C, C) CDDO-Me at T = 10 min, 26°C D) HPP-4382 at T = 10 min, 26°C

**Figure S3: Schematic reaction between CDDO-Me and GSH**

Reference

Couch RD, Browning R, Honda T, Gribble GW, Wright DL, et al. (2005) Studies on the reactivity of CDDO, a promising new chemopreventive and chemotherapeutic agent: implications for a molecular mechanism of action. Bioorg Med Chem Lett 15: 2215-2219
