# Supplementary material for: Induction of Heme Oxygenase I (HMOX1) by HPP-4382: A Novel Modulator of Bach1 Activity
Source: PLoS One. 2014 Jul 14;9(7):e101044. doi: 10.1371/journal.pone.0101044 (PMC4096395; doi:10.1371/journal.pone.0101044)
Supplement: Data S3 — Expression of Phase II genes in NHLF cells. (DOCX) [file pone.0101044.s003.docx]

Data S3: Expression of Phase II genes in NHLF cells

Gene expression was determined using the QuantiGene II system from Affymetrix using the manufacturer’s protocol. Briefly, NHLF cells were grown in 96-well CoStar™ tissue culture plates (4,000 cells per well) and treated with compounds for 5 hours in 100 µL complete medium per well. Cells were then lysed by adding 50 µL Lysis Buffer. Following the provided protocol, a portion of the RNA-containing lysate (5-10 µl) was hybridized at 54 degrees C overnight to RNA specific magnetic capture beads in the presence of blocking buffers, proteinase K and preordered mRNA probe sets specific for the genes of interest: HMOX1, NAD(P)H dehydrogenase, quinone-1 (NQO1), thioredoxin reductase-1 (TXNRD1), and GAPDH. With the aid of a magnetic plate holder, capture beads containing the hybridized mRNA were washed and incubated with a series of provided labeling probes were bound to streptavidin. The amount and intensity of the labeled beads were determined using a Luminex™ xMAP cytometric scanner (BioRad). Results were tabulated and plotted using JMP software.

**Table S3: Fold Expression of Phase II Genes in NHLF cells.** HLF cells exposed compounds for 5 hours were lysed and probed for transcription of HMOX-1, NQO1, and TXNRD1 using the QuantiGene II RNA plex. Detected RNA per well was normalized to total GAPDH in same well and shown as fold induction over DMSO.

| **Gene** | **CoPP**  **10 µM** | **CDDO-Me**  **0.3 µM** | **HPP-1014**  **10 µM** | **HPP-4382**  **3 µM** |
| --- | --- | --- | --- | --- |
| HMOX1 | 49 ± 0.09 | 37.3 ± 0.1 | 41.5 ± 0.9 | 39.0 ± 2.0 |
| NQO1 | 3.1 ± 0.04 | 1.1 ± 0.08 | 1.2 ± 0.001 | 2.2 ± 0.06 |
| TXNRD1 | 5.7 ± 0.5 | 2.0 ± 0.07 | 2.3 ± 0.06 | 2.9 ± 0.07 |
